# EVERYDAY MEMORY IN OLDER AND YOUNGER ADULTS: OUTSOURCING, SOCIALLY-DISTRIBUTED REMEMBERING & CONCEPTUALIZATION

**DOI:** 10.1093/geroni/igz038.3349

**Published:** 2019-11-08

**Authors:** Emily Lustig, Christopher Hertzog, Ann Pearman, Kirsten Reynolds, Aiman Waris

**Affiliations:** Georgia Institute of Technology, Atlanta, Georgia, United States

## Abstract

Qualitative interview data about everyday remembering within the context of older adults ages 62-83, (N= 27, M=69.5, SD=5.72) and younger adults ages 18-24 (N=29, M=21.2, SD= 1.77) lives were collected and analyzed using constructivist grounded theory methods. This study sought to compare the processes used by these individuals in their pursuit of everyday memory-demanding goals and their conceptualization of these methods. Older adults typically reported importance beliefs that guided memory-supportive behaviors for things like social engagements or medical appointments, whereas younger adults reported important information as being primarily school-related. There were major differences in the execution and conceptualization for remembering critical information. Younger adults engaged in a form of socially-distributed cognition, wherein they relied on and outsourced remembering to technology and other people via apps. Interestingly, younger adults relied on others to remind them about coursework, extra-curricular activities, and social obligations via social communication platforms (e.g. GroupMe), text messages, and shared calendar alerts. Very few of the younger adults sampled were responsible for reminding others, but relied on the social altruism of their peers who were responsible for disseminating mass reminders. Conversely, technological outsourcing was not as prevalent in the older adults interviewed and only a few shared that they received similar reminders via text or email. Of the few cases that did outsource, a small subset did, however, engage in these processes within small groups or pairs, wherein a friend or significant other reminded them about social gatherings or names but in a much smaller proportion, comparatively.

